# Effect of SiC Content on the Ablation and Oxidation Behavior of ZrB_2_-Based Ultra High Temperature Ceramic Composites

**DOI:** 10.3390/ma6051730

**Published:** 2013-04-29

**Authors:** Ping Hu, Kaixuan Gui, Yang Yang, Shun Dong, Xinghong Zhang

**Affiliations:** Science and Technology on Advanced Composites in Special Environment Laboratory, Harbin Institute of Technology, Harbin 150001, China; E-Mails: gkx89@sina.com (K.G.); 18746026280@163.com (Y.Y.); 18746046862@163.com (S.D.); zhangxh@hit.edu.cn (X.Z.)

**Keywords:** ZrB_2_, SiC, ultra high temperature ceramics (UHTCs), ablation, oxidation

## Abstract

The ablation and oxidation of ZrB_2_-based ultra high temperature ceramic (UHTC) composites containing 10%, 15% and 30% v/v SiC were tested under different heat fluxes in a high frequency plasma wind tunnel. Performance was significantly affected by the surface temperature, which was strongly dependent on the composition. Composites containing 10% SiC showed the highest surface temperature (>2300 °C) and underwent a marked degradation under both conditions. In contrast, composites with 30% SiC exhibited the lowest surface temperature (<2000 °C) and demonstrated excellent ablation resistance. The surface temperature of UHTCs in aerothermal testing was closely associated with the dynamic evolution of the surface and bulk oxide properties, especially for the change in chemical composition on the exposed surface, which was strongly dependent on the material composition and testing parameters (*i.e.*, heat flux, enthalpy, pressure and test time), and in turn affected its oxidation performance.

## 1. Introduction

Refractory metal borides such as zirconium diboride (ZrB_2_) and hafnium diboride (HfB_2_) have been commonly referred to as ultra high temperature ceramics (UHTCs), for their extremely high melting temperatures (around 3300 and 3500 K respectively) [1]. ZrB_2_ and HfB_2_ based UHTC composites represent a class of promising materials for use in extreme applications such as sharp leading edge and control surface components on hypersonic vehicles, because of their high melting point, retained strength at elevated temperatures, relatively good oxidation resistance, and dimensional stability in hypersonic flight conditions [2,3,4,5,6,7,8]. Zirconium diboride has some advantages over hafnium diboride as an aerospace material because it is lighter and less expensive while having comparable levels of oxidation resistance [4,7,8,9,10,11,12].

ZrB_2_–SiC composites are currently considered the baseline ultra high temperature ceramic composites. Indeed, varying the starting composition by changing the SiC content has given added flexibility in optimizing specific microstructure designs. Adjusting the SiC content in ZrB_2_ matrix, for instance, has proven beneficial for oxidation and ablation resistance [3,7,9,10,11,12,13,14]. Specifically, compositions containing from 10% to 30% (by volume) SiC have generally been found to be optimal in this regard. The oxidation resistance of ZrB_2_–SiC of various compositions under the same oxidizing condition has been studied [7,14]. However, these have focused primarily on static or flowing air studies using furnace testing, and few studies have evaluated their oxidation behavior in simulated hypersonic flight environments [9,10,11,12,13]. Oxidation testing results involving both furnace oxidation testing and plasma wind tunnel testing (or arc-jet testing) showed that UHTCs have a similar microstructure of the oxide scale and oxidation resistance at a comparable level of the sample surface temperature [7,8,9,10,11,12,13,14,15,16]. A high-temperature thermal protection system (TPS) intended for the leading edge and control surface components of a hypersonic vehicle will likely encounter partially dissociated air in chemical non-equilibrium with the TPS surface. This will cause a different surface temperature for samples with different compositions under the same testing conditions [17]. Unfortunately, data regarding the response of material composition on the surface temperature of UHTCs is not available. Reducing the surface temperature of the materials by changing composition may be a good way to greatly improve the material performance in hypersonic conditions, thus enabling hypersonic vehicles to operate under higher heat flux conditions. How to decrease the surface temperature is an urgent issue for aerospace applications, and much more attention should be paid to the thermal response of UHTC composites to the service environment in the future.

The purpose of this paper is to investigate the effect of SiC content on the performance of ZrB_2_ based ultra high temperature ceramic composites under different heat flux using high frequency plasma wind tunnel. The ablation resistance, oxidation behavior and thermal response of these composites is discussed.

## 2. Experimental Procedure

The samples for ablation tests were fabricated from commercial ZrB_2_ (Northwest Institute for Non-ferrous Metal Research, China) and SiC (Weifang Kaihua Micro-powder Co. Ltd., China) powders. The ZrB_2_ and SiC powders had the same purity of 99.5% and their mean particle sizes were 2 μm and 0.5 μm respectively. The powder mixtures of ZrB_2_ with 10% SiC v/v (ZS10), ZrB_2_ with 15% v/v SiC (ZS15), and ZrB_2_ with 30% v/v SiC (ZS30) were ball milled in ethanol for 8 h with hard milling media, and dried in a rotating evaporator. Milled powder was then uniaxially hot pressed in a boron nitride coated graphite die at 1950 °C for 60 min under vacuum and 30 MPa of applied pressure. All the samples have a nearly full density and the effect of porosity on the oxidation behavior can be ignored.

The samples with a Φ20 mm × 30 mm cylinder for ablation tests were cut from the billet, and all surfaces were diamond polished to a 1 μm finish then exposed to sustained high enthalpy flows using a high frequency plasma wind tunnel. Coupons were ultrasonically cleaned, successively in detergent, de-ionized water, acetone and alcohol prior to their exposure. Test specimens were mounted in graphite holders attached to a water cooled sting arm. The cold wall stagnation point heat flux was measured using a water cooled copper calorimeter installed flush with the surface of a water cooled copper probe. The performance of ZrB_2_–SiC UHTCs in subsonic high enthalpy dissociated airflows was investigated. The gas mass flow rate was 3–4 g/s. The other main testing parameters are summarized in Table 1. The experiments were carried out with a two-colour Raytek pyrometer (RAYMR1SCSF, USA), which covers a temperature range of 1000 to 3000 °C. X-ray diffraction (Rigaku, Dmax-rb, Japan), scanning electron microscopy (FEI Sirion, the Netherland), and energy dispersive spectroscopy (FEI Sirion, the Netherlands) were used to characterize the phase composition and microstructure of the surface, and the cross section of the samples after testing.

**Table 1:** Testing parameters.

Condition	Heat flux (MW/m^2^)	Enthalpy (MJ/kg)	Pressure (Kpa)
1	4.78	27.9	18
2	3.80	20.8	18

## 3. Results

**Figure 1:**
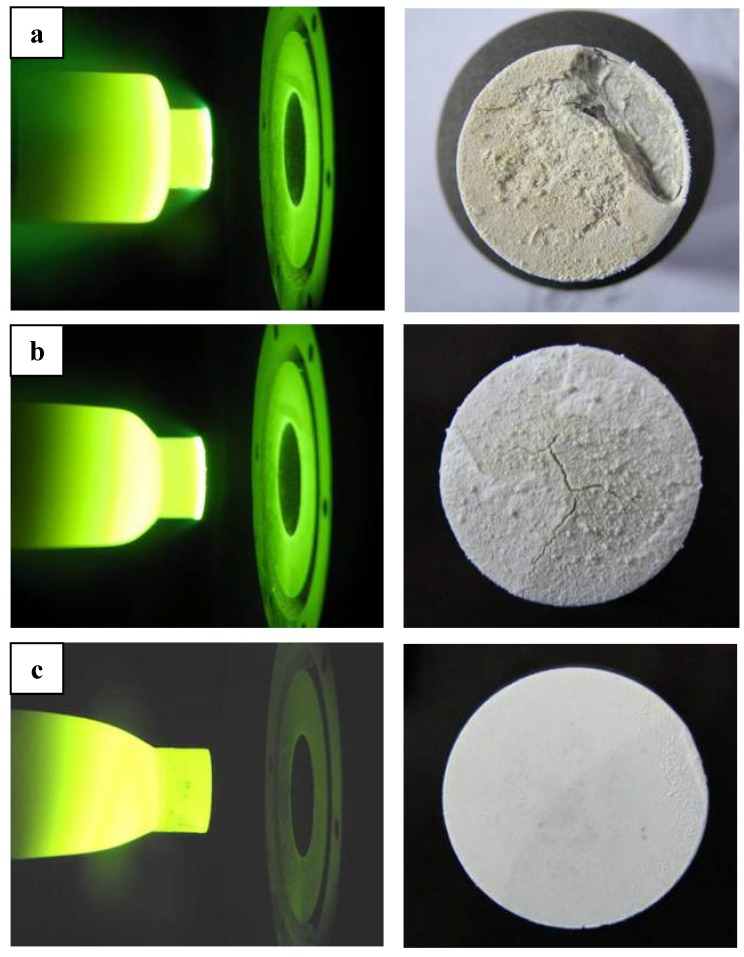
Photographs of ZrB_2_ based ultra high temperature ceramic compositesduring and after ablation tests under condition 1: (**a**) ZS10; (**b**) ZS15; (**c**) ZS30.

**Figure 2:**
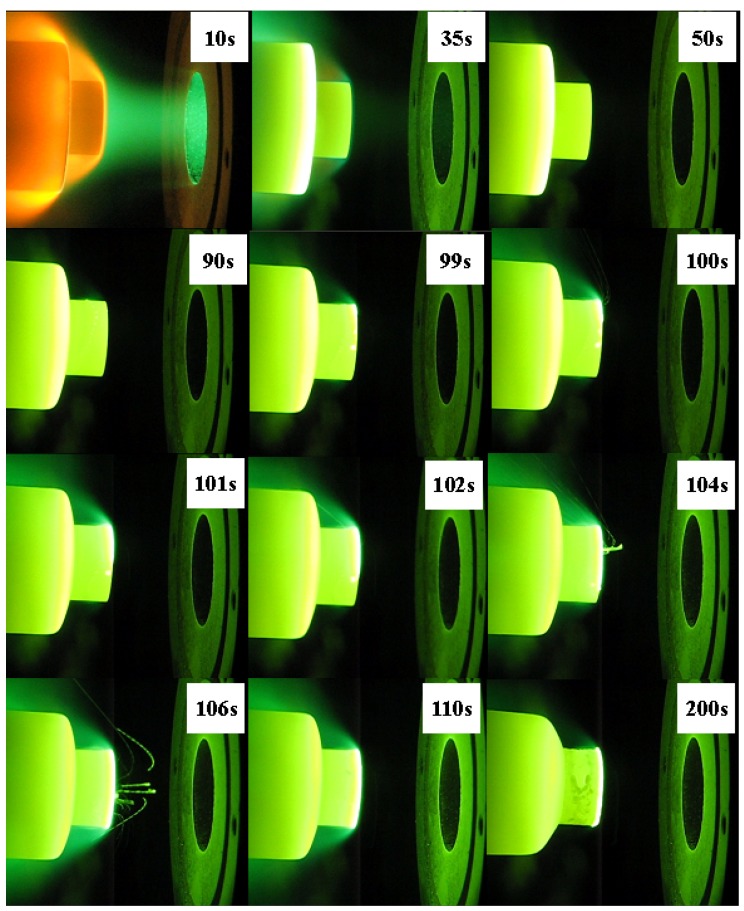
Images of ZS10 during ablation tests under condition 1.

ZS10 and ZS15 displayed significant ablation after tests under condition 1, whereas ZS30 exhibited excellent ablation resistance and configuration stability, as shown in Figure 1. Detailed ablation results are given in Table 2. The oxide scales were not adherent to the base material for ZS10 or ZS15. Rough surfaces, along with some holes and crests, were detected on these two composites, besides cracks. In contrast, a smooth surface was observed for ZS30, and the formed oxide scale was adherent to the base material. It should be noted that there was a marked difference on the surface temperature between these composites. ZS10 displayed the highest surface temperature, and the lowest surface temperature was found in ZS30. In addition, a temperature jump was detected for ZS10, as shown in Figure 2. Comparing the results of the two conditions, it appears that the surface temperatures are in good agreement and that the surface temperatures obtained for a sample under condition 2 are lower than those under the condition 1. ZS30 exhibited the lowest surface temperature in both conditions. In particular, although the heat flux in condition 1 was higher than that in condition 2, the surface temperature of ZS30 under condition 1 was still lower than that of both ZS10 and ZS15 under condition 2.

**Table 2:** Ablation results of ZrB_2_-based ultra high temperature ceramic composites.

Materials	Condition	Original mass (g)	Mass after ablation (g)	Steady state surface temperature (°C)	Ablation time (s)
ZrB_2_–10 vol % SiC	1	51.44	50.99	2450	300
ZrB_2_–15 vol % SiC	1	53.61	53.52	2320	600
ZrB_2_–30 vol % SiC	1	49.81	49.78	1960	600
ZrB_2_–10 vol % SiC	2	51.54	51.18	2330	600
ZrB_2_–15 vol % SiC	2	53.68	53.66	2250	600
ZrB_2_–30 vol % SiC	2	49.76	49.77	1900	600

**Figure 3:**
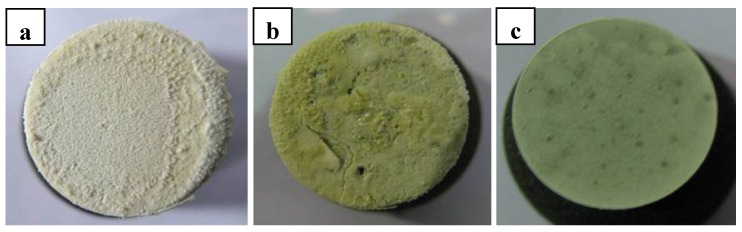
Photographs of ZrB_2_ based ultra high temperature ceramic composites after ablation tests under condition 2: (**a**) ZS10; (**b**) ZS15; (**c**) ZS30.

Figure 3 shows the photographs of ZrB_2_–SiC composites after ablation tests under condition 2. Spallation was not observed after cooling to room temperature because the heat flux was lower than that in condition 1. ZS30 also showed the best ablation and oxidation resistance among these composites under the condition 2, which is consistent with the results under condition 1. The surface colors of these composites became grayer with increasing SiC content. ZrO_2_ appeared white, while silica glass appeared dark at room temperature, meaning that the amount of the silica glass at the surface increases with increasing SiC content.

**Figure 4:**
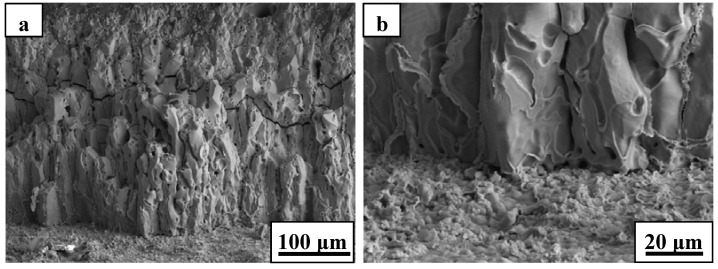
SEM micrographs of the oxide scale for ZS10 after ablation under condition 1: (**a**) low magnification; (**b**) high magnification.

**Figure 5:**
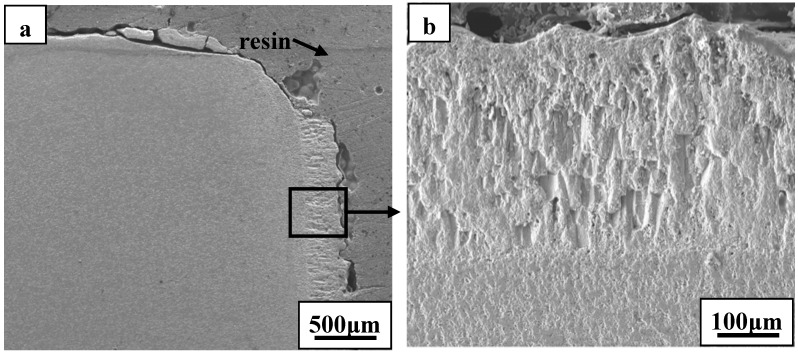
Cross-sectional morphologies of ZS10 after ablation under condition 1: (**a**) low magnification; (**b**) high magnification of the scale at the side surface.

**Figure 6:**
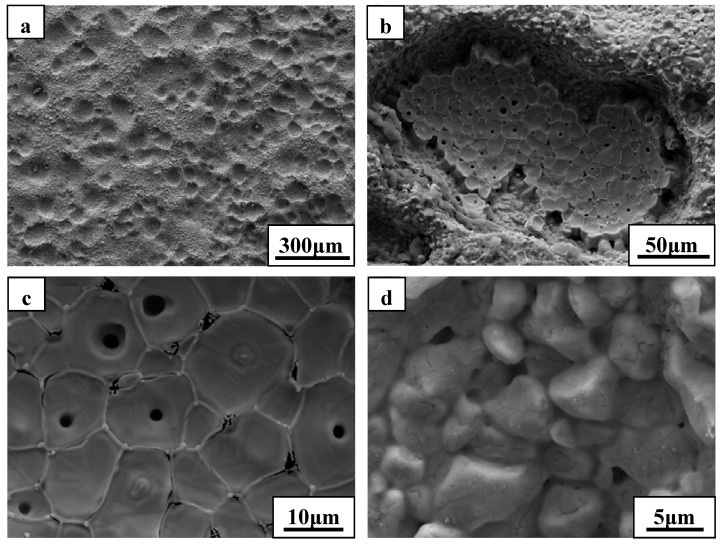
SEM micrographs of the substrate for ZS10 after ablation under condition 1: (**a**) low magnification; (**b**) , (**c**) and (**d**) are higher magnifications of (**a**).

The oxide scale consisted primarily of zirconia and showed an oriented growth during ablation testing of ZS10 under condition 1 (Figure 4). A columnar shape of the scale, containing some voids, was detected after ablation. Such observations are consistent with the furnace oxidation of these composites being at temperatures of 1800 °C and above [14,15,16,18]. The outside oxide scale easily cracked, or spalled, during cooling to room temperature, owing to the different CTEs between oxide scale and the base material, as shown in Figure 4. The phase transformation of zirconia, with an accompanying volume change, can easily lead to cracking and spalling upon cooling to room temperature. Furthermore, the formation of high pressure gas phases (e.g., B_2_O_3_, CO, and SiO) resulting from the oxidation of ZrB_2_ and SiC was responsible for the rupture of the oxide scale during the oxidation process. These are possible mechanisms to explain the scale loss that was observed. High magnification of the oxide scale showed clear evidence of gas flow, which indicated that gaseous products were escaping from the material. In addition, a large number of pores were embedded in the ZrO_2_ rich oxide scale. Such observations have not been detected in previous studies. Cross-sectional morphologies of ZS10 after ablation under condition 1 revealed that the top surface layer had flaked off, as shown in Figure 5. The total pressure in the inner oxide scale is a key factor in the mechanical stability of the oxide scale. Thermodynamic calculations indicated that at an air temperature of 2066 °C, the total pressure of B_2_O_3_ at the ZrB_2_/ZrO_2_ interface would reach 1 atm [19]. Thus, the total pressure of the interior oxide scale was higher than ambient pressure, since the surface temperature was far higher than 2066 °C and the ambient pressure was lower than 1 atm under the conditions used. The side surface scale was adherent to the base material because the temperature in this region was significantly lower than that at top surface. Figure 6 shows SEM images of the substrate in which the outside scale was lost. The surface was not smooth, and a large number of concave pits were detected. The growth of oxide scale with a cylindrical shape in the concave pit is obvious from Figure 6c. Further growth of these cylindrical grains would sinter together with the outside scale and lead to an increase of the thickness of the outside scale. Pores were commonly detected at the grain boundaries and the inner grains, as a result of the outflow of the gaseous phase products. This observation is consistent with the microstructure of the outside scale, as shown in Figure 4. Interestingly, the microstructure and morphology of the substrate showed a particle shape (Figure 6d) similar to the ZrB_2_ grains. In addition, a number of cracks were detected in the grains due to volume expansion on the conversion of ZrB_2_ to ZrO_2_. EDS analysis of the substrate layer showed that ZrB_2_ was not completely transformed to ZrO_2_ (not shown). The apparent growth of ZrO_2_ has not occurred in this region. We also found that surface of the substrate had an intact structure, but little evidence of fracture was observed. Such observations indicate that the outside scale was not effectively adherent to the substrate, which was further confirmed by the fact that the formed outside scale flaked off by itself at room temperature.

**Figure 7:**
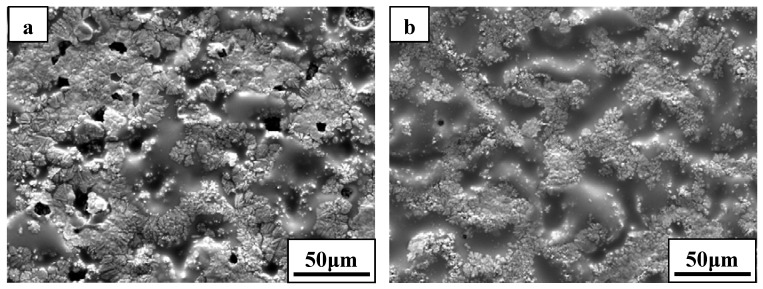
Surface morphologies of ZS30 after ablation under (**a**) condition 1 and (**b**) condition 2.

**Figure 8:**
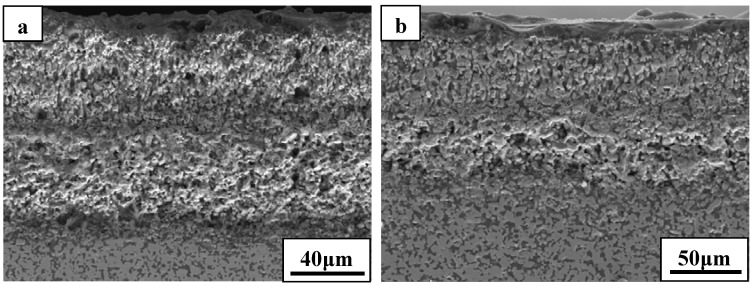
Cross-sectional morphologies of ZS30 after ablation under (**a**) condition 1 and (**b**) condition 2.

**Figure 9:**
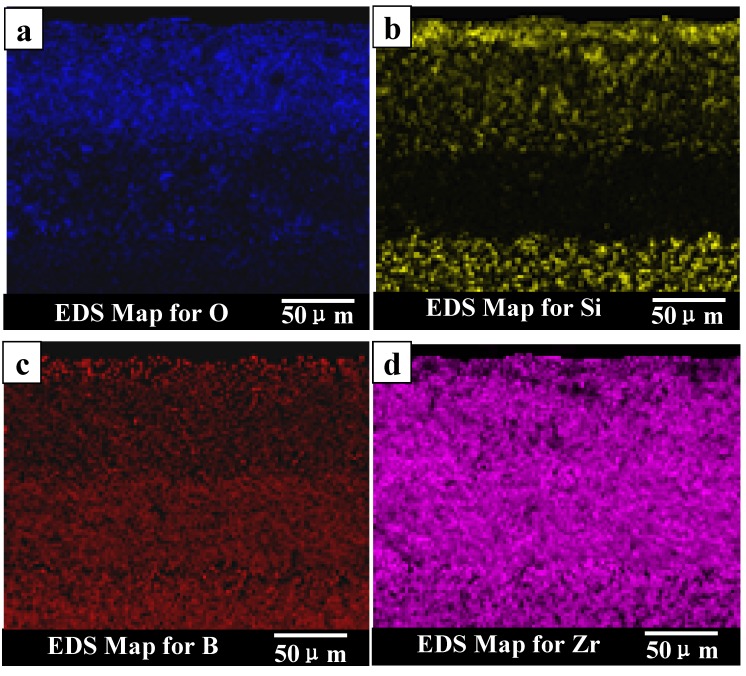
Elemental maps for the oxide scale of ZS30 after ablation under condition 1.

**Figure 10:**
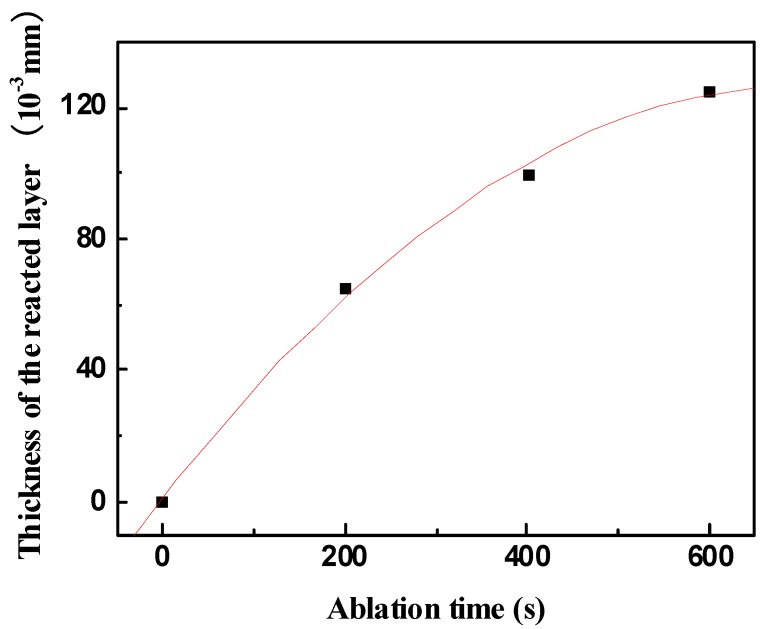
Thickness of the reacted layer *vs.* ablation time obtained under condition 1.

SEM micrograph of the exposed surface of ZS30 after ablation testing under condition 2 revealed a coherent and compact silica glass coating decorated with aggregated zirconia crystals of various sizes and shapes, as shown in Figure 7. In contrast, the surface was not smooth and contained a number of pores on the surface after ablation under condition 1. Moreover, the amount of the silica glass formed on the surface layer under condition 1 was much lower than that formed under condition 2. Such observations are attributed to the different surface temperature as a consequence of the change of the test condition. Figure 8 shows the cross-sectional morphologies of ZS30 after ablation tests under the two conditions. The oxide scale formed under condition 2 was more compact than that formed under condition 1. Moreover, the formed ZrO_2_ had not apparently changed the initial frame structure of the ZrB_2_ after ablation under condition 2. A silica rich outer layer was formed under the present condition, as shown in Figure 8. External silica scales, such as those observed in the present study, have been shown to limit the inward diffusion of oxygen, thus enhancing the resistance to oxidation [7,9]. The thickness of oxide scale for ZS30 ablated under condition 2 for 600 s was only ≈90 μm, which is significantly lower than that of ZS10 and ZS15 under the same condition. Under harsher conditions, the silica rich glass layer was not observed in the cross-section, which is consistent with surface microstructure. Silica glass became unstable and was lost by rapid vaporization in the present case. The elemental mapping by SEM-EDS throughout the oxide scale after ablation at condition 1 for ZS30 revealed the formation of a two-layer structure, as can be seen in Figure 9. The silica glass were formed and homogeneously distributed in outside scale, which effectively plugged pores and sealed ZrO_2_ boundaries leading to an enhanced oxidation resistance. A little silica glass was observed in the inner oxide scale, as a result of the active oxidation of SiC. The ZrO_2_ skeleton was continuously formed, and no evidence of cracking or spallation was detected (Figure 9d). ZrO_2_ provides an oxide skeleton without being blown away by the gas flow, leading to configurational stability of the material. To ensure good resistance, the protective condensed-phase oxide scale must be thermochemically and mechanically stable. ZS30 exhibits passive oxidation with parabolic kinetics under high heat flux, as shown in Figure 10. This indicates that the formed scale acts as an effective barrier for diffusion of oxygen to the underlying material.

## 4. Discussion

The ablation and oxidation of UHTC samples exposed to frequency plasma wind tunnel suggests that SiC content has a significant impact on the surface temperature and oxidation resistance. This can be explained by the evolution of the surface composition and microstructure, which is the main reason for the surface temperature difference. Unfortunately, the major factor that accounts for the temperature difference for UHTCs with different compositions under the same test is not well understood. While ZrB_2_–SiC ultra high temperature ceramic composites have been studied extensively in the space field and have been fully characterized in their base thermo-mechanical properties, little attention has been paid to their radiative and surface catalytic behavior at ultra high temperatures. Nonetheless, emissivity and surface catalycity represent key parameters for space re-entry thermal protection system applications. When a space vehicle flies in a very high Mach number, a very strong bow shock is formed in front of the body, and the dissociation of air occurs in the shock layer. Some of the dissociated atomic nitrogen and atomic oxygen recombine on the surface. Since recombination processes are exothermic (498 kJ released per mole of O_2_ formed, 945 kJ per mole of N_2_, and 415 kJ per mole of NO) [20,21] part of the released energy is transferred to the surface of materials with the consequent increasing of their surface temperature [22,23,24]. The influence of SiC content on the surface temperature has not been recognized in the previous literature. Marianne Balat-Pichelin *et al.* have studied the influence of the microstructure of the material in the case of silica and alumina, and indicated that the surface morphology, and particularly roughness, play a very important role in the atomic oxygen recombination coefficient γ_0_, which increases with increasing surface roughness [25]. Significant swelling of the oxide scale occurred during the oxidation of low SiC content material, due to the volume expansion as a result of ZrB_2_ oxidation. This led to the formation of a rough surface layer during oxidation, which may have caused the increment of the atomic recombination coefficient, resulting in an increase of surface temperature. It has been reported that silica glass has very weak catalytic activity and high radiative efficiency compared with other materials [26,27]. Therefore, the existence of silica glass on the surface would lead to a decrease in surface temperature. High content of silica glass was observed on the surface of ZS30 after ablation, which would account for it having the lowest surface temperature among all the materials.

The different oxidation rate of these materials under the same condition may be another reason for the surface temperature difference, since the oxidation processes are also exothermic. Surface oxidation processes that transform ZrB_2_ and SiC into condensed and volatile oxides are all exothermic, and release more energy per oxygen atom consumed than catalytic recombination to molecular oxygen [17]. Thus, the effect of the oxidation on the surface temperature cannot be neglected, and the oxidation rate also plays an important role in the heat formation that led to an increase of surface temperature. It should be noted that the difference among the surface temperatures of the UHTC specimens with low and high SiC content was small during initial heating and until a steady state was reached. Obviously, the oxidation rate will decrease, and at least not increase with increasing time, when the sample reaches the equilibrium state, resulting in a reduction of heat formation. From the exothermic point of view, the oxidation will not lead to the further increase of the surface temperature when the UHTC specimens achieve a steady state. In fact, a large temperature jump, with a magnitude of 300–500 °C, was found in UHTCs containing low SiC, resulting in higher surface temperatures. Therefore, the different oxidation rates of ZrB_2_–SiC composites are not the main factor for the large surface temperature differences, because the oxidation rates of these materials were all relatively low before the temperature jump, under the conditions used in this study. Of course, the heat contribution to the heat flux coming from the material oxidation should be taken into account where the material has a very high oxidation rate.

Similar phenomena was also detected by Marschall J. *et al.* who concluded that a temperature jump of ZrB_2_–SiC UHTCs was associated with a transition in surface chemistry involving the Si-containing compounds (the silica-rich oxide scale and the silica former SiC) and is likely related to the passive-to-active oxidation transition observed for other SiC-containing composites in aerothermal test environments [17]. The total emittance values for the oxides of ZrB_2_–SiC UHTCs are shown to change slightly over a large temperature range [9]. So, the change in emittance of the oxide scale is not sufficient to be the driving force for the large temperature jump, according to thermal radiation equilibrium assessment. Moreover, the total emittance for systems of similar materials has been shown to change slightly before and after the temperature jump occurs [17]. Therefore, the change in emittance during the ablation process may not contribute to the large surface temperature difference in the present study.

According to the discussion above, the possible main reasons for the large surface temperature difference are the increase of the chemical heating contribution, the decrease of the heat conduction into the interior, or both—as a consequence of the dynamic evolution of the surface and bulk oxide properties. The condensed phases for the oxide scale of the ZrB_2_–SiC composites are ZrO_2_ and SiO_2_ under conditions 1 and 2 respectively. During the initial heating process in aerothermal test environments, SiC underwent passive oxidation, resulting in the formation of SiO_2_ that was mostly distributed on the surface, since the temperature has not reached the passive-to-active oxidation transition. Following that, active oxidation of SiC would occur with the prolongation of the heating time, accompanied by a further increase of the surface temperature up to a steady state. It should be noted that the steady state temperature is much higher than that of the passive-to-active oxidation transition for SiC in the case of present tests [19]. Obviously, the active oxidation of SiC has minor effect on the change of the surface composition when the specimen achieved the steady state. Therefore, the temperature jump is not directly associated with a passive-to-active transition of SiC, since the active oxidation of SiC for ZS30 were also observed under both conditions, as shown in Figure 7, where no large temperature jump occurred. Note that silica volatilization becomes significant as temperature increases up to a steady state temperature (~1900 °C). Consequently, the amount of silica in the surface layer decreases gradually with the prolongation of the testing time which would lead to an increase of catalytic efficiencies for oxygen recombination, because silica surfaces are known to have relatively low catalytic efficiencies for oxygen recombination compared to zirconia surfaces [28,29]. Therefore, the sample will experience a higher chemical heating contribution to the heat flux for a given free stream condition, especially under high enthalpy conditions. A pronounced increment in surface temperature within a short time interval will be observed when the surface silica has reduced to a certain level. The thickness of oxide scale increased with increasing time before the temperature jump, and hence the heat conduction into the interior decreases because of lower heat conductivity of oxide scale, compared to that of the base material. In addition, heat conductivity of the oxide scale was also influenced by microstructural evolution during the oxidation process. These will lead to a rise in surface temperature. However, the oxide scale is adherent to the base material, and passive oxidation of UHTCs was detected before a temperature jump in the present case, and its heat conductivity changed gradually, which would not have caused a large temperature jump in a short time. To verify the strong influence of the composition on the surface temperature, ablation behavior of the sample containing AlN was carried out as a comparison. The surface temperature of this composite is significantly higher (by ~700 °C) than those without AlN since the oxide of AlN (alumina) is a well-known high catalycity material [27,30]. Therefore, the different chemical heating of the surface oxide layer contributes mostly to the large temperature jump, which depends on the material composition and testing conditions.

In the present study, the temperature jump always occurred at the edge of the sample and then propagated to other areas, especially around the sample periphery (as shown in Figure 2), because the heat flux and shear force at the edge are higher than those in the center for the cylinder shape model. This might cause the fast loss of silica around the sample periphery, leading to a significant increase in surface temperature as a result of substantial increases in the chemical heating component that delivered to the surface.

Generally, the temperature jump occurred at high temperatures, above 1900 °C, under the gas pressure used in this study. The exact value for temperature was hard to measure during the testing because of experimental constraints. The related test results indicate that the variation of pressure in a certain range has a minor effect on the transition of temperature jump. Additionally, the time for the jump was dependent on the initial steady state temperature, and decreased with the increasing temperature, since the change of the surface composition at higher temperature became faster, so played an important role in the chemical heating contribution. The temperature jump always occurred at high temperature (e.g., >1900 °C), in several minutes, and the time to trigger the spontaneous temperature jump was shortened as the surface temperature increased because the loss of silica increased rapidly with increasing temperature. In contrast, the temperature jump did not occur at lower temperatures, since the oxide scale was always covered by a stable silica glass layer during the entire testing period. Meanwhile, small temperature fluctuation and good reproducibility were observed on a series of sample tests at low heat flux condition, *i.e.*, with a lower surface temperature. Furthermore, the time to initiate the spontaneous temperature jump was affected by the thickness of the silica glass. The surface scale containing silica with a higher thickness needed more time to consume. The steady state time during the testing was correlated to the loss rate of SiO_2_ and the amount of pre-existing SiO_2_ at the beginning of steady state. Lower SiC content produced less SiO_2_ during the initial heating process, leading to the temperature jump and a higher surface temperature in the current tests. The different steady state surface temperatures achieved for the different compositions were related to the steady state energy balance at the surface, as determined by radiation, catalysis, oxidation, and convection. The temperature jump is a transient phenomenon related to a transition in Si species related surface chemistry that changes the surface heating rate under constant free stream conditions.

The molten oxide was found after the temperature jump (shown in Figure 2) and then quickly spread to the entire sample surface. The temperature increase of the surface layer also induced the temperature increase of the interior reaction region. This in turn led to formation of high pressure gas phases (e.g., B_2_O_3_, CO, and SiO) within the oxide layer, causing the rupture of the oxide scale. The ejection of molten oxide was detected in video images. A large number of pores were generated after the ejection of molten oxide, enabling the free release of the formed gas phase products, and then sample surface reached a new steady state. Poor adherence and decreased thermal contact of exterior scale to the underlying material may contribute to large temperature gradients through the oxide scale after the temperature jump. The outside scale acted as a thermal barrier coating, and this caused the decrease of the oxidation reaction rate of the inner material. Therefore, the outside scale provides an efficacious protective ablation barrier, although it is not very effective in limiting the inward diffusion of oxygen into the inner bulk.

The performance of ZrB_2_-based UHTC composites was significantly affected by the surface temperature, which seems associated with surface and bulk oxide layer properties that strongly depend on the material composition and testing conditions. The microstructural evolution of the oxide scale and the change of surface chemistry as the testing time proceeded caused the temperature to rise sharply as a result of the thermal response change under high heat flux condition, especially with high enthalpy. The ablation and oxidation properties of UHTCs are directly correlated with the given service environment, as well as the material itself. The furnace oxidation testing method is cheaper compared with other methods, such as arc-jet testing and high frequency plasma testing, and allows the sensitive control of temperature, oxidation time, oxidizing atmosphere, and pressure, whereas it cannot reflect the thermal response of the service environment on the material. It is worth noting that the oxidation performance of UHTCs is strongly dependent on the specific oxidizing environment. For example, the addition of Ta-containing compounds is beneficial to the oxidation resistance of UHTCs at temperatures below 1700 °C, whereas it has a detrimental effect on the oxidation resistance at temperatures of 1800 °C or above [31]. Oxidation testing results involving both furnace oxidation testing and plasma wind tunnel testing (or arc-jet testing) indicated that the oxidation resistance of UHTCs is strongly dependent on the oxidation temperature [8,9,10,11,12,13,14,15,16]. The present results showed that the oxidation performance of UHTCs can be improved by reducing the surface temperature of the material, based on the composition optimization in the given oxidizing environment. However, the effect of material composition on thermal response under a given aerodynamic heating condition is unclear and additional testing will be required in this respect in the future. Therefore, it is advisable to balance the thermal response and oxidation resistance of the UHTCs against temperature when choosing materials for extreme oxidizing environment.

## 5. Summary

ZS10 displayed significant ablation after tests under the two conditions, whereas ZS30 exhibited an excellent ablation resistance and configurational stability. The significant difference in ablation behavior was mostly attributed to different surface temperature. ZS10 showed the highest surface temperature (>2300 °C) while ZS30 exhibited the lowest surface temperature (<2000 °C) in both conditions. The marked difference in surface temperature was presumably caused by the different chemical component of heat flux delivered to the surface, which strongly depended on the material composition and aerothermal heating environment. This in turn affected its oxidation performance. It is advisable to balance the thermal response and oxidation resistance of the UHTCs against temperature when choosing materials for extreme oxidizing environment.
